# Factors Associated with Development of High-Grade Squamous Intraepithelial Lesions of the Uterine Cervix in Women Younger than 30 Years

**DOI:** 10.31557/APJCP.2019.20.4.1031

**Published:** 2019

**Authors:** Jongpeeti Wudtisan, Charuwan Tantipalakorn, Kittipat Charoenkwan, Rung-Aroon Sreshthaputra, Jatupol Srisomboon

**Affiliations:** *Department of Obstetrics and Gynecology, Faculty of Medicine, Chiang Mai University, Chiang Mai, Thailand. *

**Keywords:** High-grade squamous intraepithelial lesions, risk factors, younger women, uterine cervix

## Abstract

**Objective:**

To determine the factors associated with the increased risk of developing high**-**grade squamous intraepithelial lesions (HSIL) of the uterine cervix in women younger than 30 years compared with those aged ≥ 30 years who also had HSIL.

**Methods:**

Patients with HSIL who underwent loop electrosurgical excision procedure (LEEP) between January 2006 and July 2017 at Chiang Mai University Hospital were retrospectively reviewed. We analyzed the factors associated with the development of HSIL by comparing two age groups between women aged < 30 years and those aged ≥ 30 years. The factors analyzed included the well-recognized risk factors for cervical cancer, i.e. age at sexual debut, number of sexual partners, use of oral contraceptive (OC) pills, smoking history, sexually transmitted diseases and HIV status. Univariate and multivariate logistic regressions were used to assess factors associated with the increased risk of developing HSIL in women younger than 30 years compared with those aged ≥ 30 years.

**Results:**

During the study period, there were 345 patients with HSIL, 30 were < 30 years (case group) and 315 aged ≥ 30 years (control group). By multivariate analyses , early sexual debut(OR, 2.86; 95% CI, 1.01-8.13; P=0.047), multiple sexual partners (OR, 2.94; 95% CI, 1.23-7.02; P=0.015), history of genital warts (OR, 20.46; 95% CI, 2.27-183.72; P=0.007) and history of smoking (OR, 2.95; 95% CI, 1.10-7.93; P=0.032) were significantly associated with the development of HSIL in women younger than 30 years when compared with those aged ≥ 30 years. The OC use, HIV status and underlying diseases were not significantly different in both groups.

**Conclusion:**

Early age at sexual debut, multiple sexual partners, history of genital warts and smoking are significant risk factors for developing HSIL in women younger than 30 years. Cervical cancer screening should be considered in young women with such factors.

## Introduction

Cervical cancer is the second most commonly diagnosed cancer after breast cancer and the third leading cause of cancer-related death in developing countries. In Thailand, cervical cancer is the second most common cancer in women with an estimated 10,000 new cases and 5,200 deaths in 2012 (Torre et al., 2015). From the report of Public Health Ministry of Thailand in 2015, the age-standardized incidence rate of cervical cancer in Thailand and Chiang Mai are 14.4 and 17.4 per 100,000 women per year, respectively (Imsamran et al., 2015).

The most important determinants for development of cervical cancer is human papillomavirus (HPV) infection especially the high-risk strains, type 16 and 18 which cause approximately 70% of cervical cancer and 50% of its precursors (Kahn, 2009). Factors that increase the risk of developing cervical cancer are those that increase the exposure to HPV infection or facilitate HPV persistence, i.e. early onset of sexual activity, multiple sexual partners, high parity, low immunocompetence, oral contraceptive use and cigarette smoking (Appleby et al., 2007; Cancer, 2007; Klumb et al., 2010; Ajah et al., 2014). A cross-sectional study showed that age at sexual debut of 9 – 15 years increased the risk of HPV infection (Giuliano et al., 1999). The number of sexual partners more than 5 in a life time is independently associated with cervical HPV infection with an odds ratio of 3.5 (Remschmidt et al., 2013). 

HPV infection is highly prevalent in sexually active women particularly in young women. However, most HPV infections in younger women spontaneously clear within 8 – 24 months (Ho et al., 1998). Although the prevalence of cervical HPV infection decreases after the age of 30 years, however, the risk of HPV persistence increases with age. Development of precancerous cervical lesions before the age of 30 years is quite uncommon. Cervical cancer screening is accordingly, recommended in women after the age of 30 years with the aim to detect precancerous lesion, i.e. high-grade squamous intraepithelial lesions (HSIL) that can be treated before progressing to cervical cancer.

In 2013, World Health Organization (WHO) has recommended to start screening with Pap cytology or visual Inspection with acetic acid (VIA) in women 30 years of age and older because of their higher risk of cervical cancer. In women who test negative on VIA or cytology, the screening interval for repeat screening should be every 3-5 years (WHO, 2013). In Thailand, the Ministry of Public Health has recommended to start cervical cancer screening with Pap cytology in women aged 30 years at 5 years interval until the age of 60 years (Wilailak and Lertchaipattanakul, 2016). However, there is a proportion of women who develop HSIL before the age of 30 years. We were interested in exploring what factors increase the risk of developing HSIL in women younger than 30 years in order to identify women at risk to have earlier screening before the age of recommendation. Accordingly, this study was conducted to evaluate the factors associated with the increased risk of developing HSIL in women aged less than 30 years compared with those aged ≥ 30 years who also had HSIL.

## Materials and Methods

After approval of the Research Ethics Committee of Chiang Mai University Hospital, women who underwent loop electrosurgical excision procedure (LEEP) for pathological HSIL of the uterine cervix at Chiang Mai University Hospital between January 1, 2006 and July 31, 2017 were retrospectively reviewed. The data resources for review were medical records of the colposcopy clinic of Obstetrics and Gynecology Department and the computerized database of Chiang Mai University Hospital. The patient characteristics and treatment details were reviewed, including the cervical cancer risk factors, i.e. age at first sexual relation, number of sexual partners, parity, current use of contraception either oral pills or non-oral contraception, history of smoking, HIV status, history of sexually transmitted diseases (STD) and underlying diseases. Multiple sexual partners was defined as number of partners ≥2.

The inclusion criteria were the women who had pathological diagnosis of HSIL obtained from LEEP. The women who had history of gynecologic cancer or hysterectomy for malignant gynecologic disease, incomplete medical record and pregnancy were excluded. The main outcome was to determine the risk factors associated with the development of HSIL in women aged less than 30 years compared with those aged 30 years or more. The descriptive data were presented as percentage / range or means + SD, as appropriate. The statistical analysis was performed using SPSS version 21.0 (IBM Corp. Released 2012; IBM SPSS Statistics for Windows, Version 21.0. Armonk, NY: USA). Univariate analysis was performed to examine the association between clinical risk factors and development of HSIL. Relevant odd ratios (OR) and 95% confidence intervals (95%CI) were calculated. The p-value < 0.05 was considered significant. The factors with the p-value of < 0.05 from the univariate analysis were further evaluated in multivariate analysis. 

## Results

*During the study period, 781 patients underwent LEEP for HSIL of the uterine cervix*

**Figure 1:**
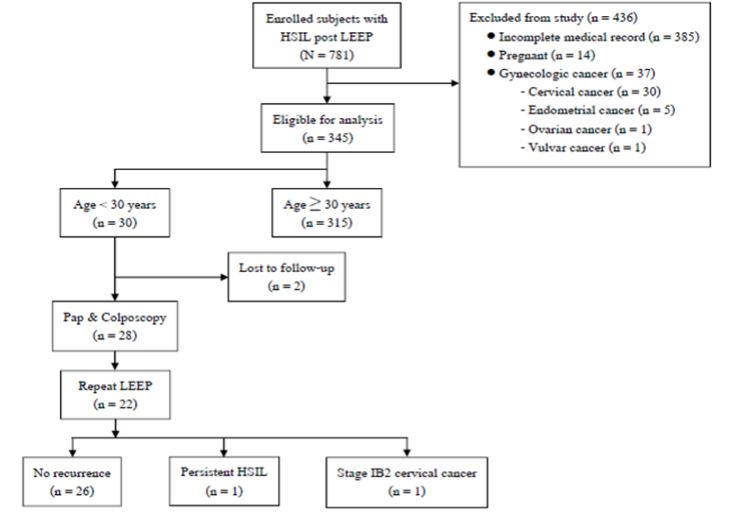
Flowchart of the Study Population

64(8.2%) were younger than 30 years. Of these, 436 patients were excluded due to incomplete medical records (385), pregnancy (14) and history of gynecologic malignancy (37) as shown in Figure 1. Finally, a total of 345 patients were enrolled in this study. Among the 345 patients with HSIL, 30 were younger than 30 years (case group) and 315 aged ≥30 years (control group). The mean ages of the patients in the case and the control groups were 26.5 years (range: 16-29 years) and 48.3 years (range: 31-79 years), respectively. The mean life-time sexual partners was 2.6(range: 1-10) in the case group and was 1.5 (range:1-6) in the control group. The mean ages at first sexual activity in the case and the control groups were 18.7 years (range: 13-29 years) and 21.1 years (13-35 years), respectively. 

The patient characteristics of both groups and univariate analysis for risk factors associated with the development of HSIL are shown in Table 1. The case group, when compared with control group had significant difference in the factors of early sexual debut (<21 years), multiple sexual partners (≥2), history of genital warts and cigarette smoking. In multivariate analysis, early sexual debut (OR, 2.86; 95% CI, 1.01-8.13; P=0.047), multiple sexual partners (OR, 2.94; 95% CI, 1.23-7.02; P=0.015), history of genital warts (OR, 20.46; 95% CI, 2.27-183.72; P=0.007) and history of smoking (OR, 2.95; 95% CI, 1.10-7.93; P=0.032) were significantly associated with the development of HSIL in women younger than 30 years (Table 2). Oral contraceptive use, HIV status and underlying diseases were not significantly different in both groups.

After LEEP in the 30 patients, 2 were lost to follow up. The remaining 28 patients were followed with Pap cytology and colposcopy at 6-months interval. Of these, 22 patients underwent repeat LEEP for abnormal cervical cytology and 18 had HSIL and 3 had LSIL. The remaining 1 had stage IB2 cervical cancer and were treated with concurrent chemoradiation. The last visits of the 28 patients showed that 26 had no recurrence, 1 had persistent HSIL and underwent simple hysterectomy, the remaining 1 with stage IB2 cervical cancer was under follow-up after chemoradiation.

## Discussion

Our study showed that among women with a diagnosis of HSIL of the uterine cervix, the significant risk factors of developing these lesions in women younger than 30 years when compared with those aged ≥ 30 years are early sexual debut, multiple sexual partners, history of genital warts and smoking. Numerous factors have been recognized as increased risk for cervical cancer including young age at first sexual intercourse, cigarette smoking, multiple lifetime sexual partners, high parity, increasing duration of contraceptive pill use, younger age at first full-term pregnancy, history of genital warts, low socio-economic status and immunosuppression (Giuliano et al., 1999; Cancer, 2007; Remschmidt et al., 2013; Zhu et al., 2015). In fact, these factors indicate the increased risk of exposure to oncogenic HPV infection or having compromised immune response to HPV infection, i.e. multiple sexual partners, young age at sexual debut, cigarette smoking, immunosuppression and previous treatment for genital warts. HPV infection was highly prevalent in women aged under 30 years (Stuardo et al., 2012). Women with history of high-risk HPV infection had an increased risk of developing HSIL in the future (Giuliano et al., 2004).

We were interested in exploring the factors influencing the development of HSIL in women younger than 30 years comparing with those aged ≥ 30 years because these young women were not included in the recommendation for routine cervical cancer screening in Thailand. We did not aim to compare the factors with women of the same age group who did not have HSIL since these factors have already been recognized as risk factors for cervical cancer. In the total of 781 women who were diagnosed with HSIL of the uterine cervix in the present study, only 64 (8.2%) were younger than 30 years. The number of HSIL cases in younger women may be underestimated since most were detected from opportunistic screening. In the ATHENA study enrolling 42,209 women aged ≥25 years in the U.S. to evaluate the performance of HPV testing as the primary screening for cervical cancer, pathological HSIL was detected in 587 women, of whom 210 (35.8%) occurred in women aged 25–29 years, despite accounting for only 16.3% of all study population. Of interest, among 347 women with CIN3 or more severe lesions, 119 (34.3%) were younger than 30 years (Wright et al., 2015). Such findings that over one-third of precancerous cervical lesions occurred in young women imply that these lesions can be detected if young women at risk are identified and appropriately screened.

In our study, age at first sexual relation of less than 21 years was significantly associated with the development of HSIL in women aged less than 30 years. The mean ages at first sexual activity seemed to be lower in younger women with HSIL (18.7 years) compared with that in older women (21.2 years) who also had HSIL. Although early age of sexual activity increases the risk of HPV acquisition, previous study noted that the age at first sexual intercourse of less than 17 year was not associated with developing HSIL in women aged 20-31 years (Remschmidt et al., 2013). 

Among the women with HSIL, the number of sexual partners ≥ 2 was significantly associated with the development of HSIL in the present study. The mean life-time sexual partners in younger women (2.6) appeared to be higher than that in the older group (1.5). This is also consistent with the previous report noting that more than 5 sexual partners was significantly correlated with HSIL in women age 20-31 years with the odds ratio (OR) of 5.1 (95% CI, 2.1-12.5;P<0.001) (Remschmidt et al., 2013). The number of sexual partners more than 5 were also remarkably linked to high-risk HPV infection (Giuliano et al., 1999). It can be concluded that the increase in number of sexual partners reflected the increased probability of acquiring HPV infection.

History of sexually transmitted infections, e.g. Chlamydia trachomatis and genital herpes is epidemiologically recognized as co-factors of cervical carcinogenesis. This factor just reflects the higher chance of acquiring other genital infections especially HPV infection including genital warts. Our multivariate analysis showed that history of genital warts were significantly associated with the development of HSIL in women younger than 30 years with the OR of 20.46 (95% CI, 2.27-183.72;P=0.007) when compared with women aged ≥ 30 years. This finding corresponds with the previous report that genital warts may be epidemiological co-factors in the development of HSIL (OR,5.7; 95% CI, 1.2-27.6;P=0.03) (Remschmidt et al., 2013).

Smoking was significantly correlated with development of HSIL in younger women when compared with the older group in our study. Previous study showed that current smoking was associated with a significantly increased risk of squamous cell carcinoma but not adenocarcinoma (Cancer, 2007). On the contrary, other studies noted that current smoking was not associated with HSIL in women aged 20-31 years (Remschmidt et al., 2013) and was not significantly associated with the occurrence of abnormal cytology (Stuardo et al., 2012).

**Table 1:** Patient Characteristics and Univariate Analysis for Risk of Developing HSIL in Women Aged < 30 Years versus Women Aged ≥30 Years

Risk factors	Number/Total (%)		P‐value
	Case (age < 30years)	Control (age ≥ 30years)	
Age at sexual debut			0.006
<21yr	25/30 (83.3%)	181/315 (57.5%)	
≥21yr	5/30 (16.7%)	134/315 (42.5%)	
Number of sexual partners			0.001
1	10/30 (33.3%)	109/315 (34.6%)	
≥2	20/30 (66.7%)	206/315 (65.4%)	
Current contraception			0.311
Oral contraceptives	9/30 (30%)	69/315 (21.9%)	
Non‐oral contraceptives	21/30(70%)	246/315 (78.1%)	
HIV status			0.978
Negative	27/30(90%)	284/315 (90.2%)	
Positive	3/30 (10%)	31/315 (9.8%)	
History of genital warts			<0.001
Yes	3/29 (10.3%)	2/312 (0.6%)	
No	26/29 (89.7%)	310/312 (99.4%)	
History of smoking			0.001
Yes	8/30 (26.7%)	26/315 (8.3%)	
No	22/30 (73.3%)	289/315 (91.7%)	
Underlying disease			0.25
Yes	7/30 (23.3%)	105/312 (33.7%)	
No	23/30 (76.7%)	207/312 (66.3%)	

HIV, Human immunodeficiency virus

**Table 2:** Multivariate Analysis for Risk of Developing HSIL in Women Aged < 30 Years versus Women Aged ≥30 Years

Risk factors	OR (95%CI)	P value
Early age at sexual debut	2.86 (1.01-8.13)	0.047
Multiple sexual partners ^1^	2.94 (1.23-7.02)	0.015
History of genital warts	20.46 (2.27-183.72)	0.007
History of smoking	2.95 (1.10-7.93)	0.032

^1^, Numbers of partners ≥2

The use of oral contraceptives (OC) was not associated with a significant increase in the risk of developing HSIL in younger women comparing with the older ones in the present study. However, the data on the duration of OC use were not available. The correlation of HSIL and cervical cancer with the OC use is not entirely consistent due to heterogeneity of the studies. The OC use of less than 5 years was not related to the risk of cervical cancer (OR,0.77; 95% CI,0.46–1.29) but it increased significantly for 5–9 years of use (OR , 2.72; 95% CI,1.36– 5.46) and for ≥10 years of use (OR,4.48; 95% CI,2.24–9.36) (Bosch and de Sanjose, 2007). Another study noted that the relative risk of cervical cancer increased in the current users of OC and declined after the cessation of OC use (Appleby et al., 2007). Since persistent HPV infection is essential for cervical carcinogenesis, thus, the association between OC use and the risk of cervical cancer should be studied in the relevant population, i.e. HPV-positive women. A pooled data of multicenter case- control study assessing the correlation of OC use and cervical cancer in HPV-positive women noted that ever use of OC was not significantly associated with an increase in cervical cancer (OR, 1.29; CI, 0.88–1.91). However, duration of OC use was significantly associated with the development of cervical cancer, i.e. HPV-positive women who used OC for 5 to 9 years (OR, 2.82; CI, 1.46–5.42) and ≥10 years (OR, 4.03; CI, 2.09–8.02) had a significant increase in the risk of cervical cancer compared with never users. The risk did not increase in women who used OC for less than 5 years (Moreno et al., 2002).

HIV status was not significantly associated with the development of HSIL in women younger than 30 years when compared with the older ones in this study. This may be caused by small number of cases in the younger group. In general, the risk of HPV infection is higher in HIV-positive women than in women who are HIV-negative. In a study involving 1778 HIV-positive and 500 HIV-negative women, it was found that 63% of the HIV- positive women tested positive to HPV DNA while only 30% of the HIV-negative participants tested positive (Palefsky et al., 1999). HIV-positive women have been reported to have 7 times more incidence of cervical cancer than their HIV-negative counterparts (Abraham et al., 2013).

The strength of the present study was the inclusion of patients who were treated at a single institution and all pathologic specimens were examined by expert gynecologic pathologists. However, certain limitations exist. As the design of this study was retrospective, some epidemiologic data were no available, e.g. the data of sexual partners (partners with multiple sexual partner, circumcision or known HPV infection), socioeconomic status, results of the HPV testing and the patients’ body mass index. Secondly, this study contained a relatively small sample size due to the rarity of this disease in young women. In addition, cervical cancer screening in women younger than 30 years in Thailand is not routinely recommended in the national policy. Therefore, younger women with precancerous cervical lesions may not be detected. Clinical implication of the findings in this study is that cervical cancer screening should also be provided to women younger than 30 years who have multiple sexual partners, early sexual debut, history of genital warts or smoking although they are not included in the routine screening program.

In conclusion, among women with pathological diagnosis of HSIL, early sexual debut, multiple sexual partners, history of genital warts and smoking are significant risk factors for developing HSIL in women younger than 30 years. Cervical cancer screening should be considered in young women with such factors.

## Conflict of Interest

The authors have no conflict of interest to disclose.
